# Conformational modulation of influenza virus hemagglutinin: characterization and *in vivo* efficacy of monomeric form

**DOI:** 10.1038/s41598-017-08021-x

**Published:** 2017-08-08

**Authors:** Jong Hyeon Seok, Jeongwon Kim, Dan Bi Lee, Ki Joon Cho, Ji-Hye Lee, Garam Bae, Mi Sook Chung, Kyung Hyun Kim

**Affiliations:** 1 Department of Biotechnology & Bioinformatics, Korea University, Sejong, 30019 Korea; 2 Department of Food and Nutrition, Duksung Women’s University, Seoul, 01369 Korea; 3 Antibody Engineering Team, Mogam Institute, Yongin Kyunggi, 16924 Korea

## Abstract

Mutational changes that mostly occur at the head region of hemagglutinin (HA) lead to the emergence of new epidemic influenza viruses, whereas HA antigens have been modified to generate broadly neutralizing antibodies toward highly conserved epitopes in the HA stem. Interestingly, a recent analysis of serum antibody repertoires showed that broadly neutralizing antibodies bind to HA monomer at a conserved region occluded at the intermonomer interface of HA trimer and confer protection in animal models. We showed previously that the recombinant HA ectodomain from a pandemic strain A/Korea/01/2009 was monomeric in solution and crystal structure. In order to examine the potential antigenicity of a monomeric form, we designed HA monomer that incorporates mutations to destabilize trimer conformations. Starting with the HA trimer from a seasonal strain A/Thailand/CU44/2006, mutations were introduced at the intermonomer interface, Ser199 of HA1 and Gly47, Arg75, Phe88, Val91, and Arg106 of HA2. Two mutants, F88E and V91W, were characterized to form a monomer and their double mutant F88E/V91W monomer was selected as an antigen. Animal studies showed that the HA monomer induced protective immunity *in vivo*, comparable to the trimer, albeit low antibody titers in sera.

## Introduction

Influenza A viruses are divided into subtypes on the basis of differences in the antigenicity of surface proteins, hemagglutinin (HA) and neuraminidase (NA). Only three HA (H1, H2 and H3) and two NA (N1 and N2) subtypes have adapted to humans thus far to produce the past pandemic influenza A viruses, including the 2009 pandemic H1N1 (pH1N1) influenza virus, which originated from nonhuman host animals^1–4^. The pH1N1 viruses replicated more extensively in the respiratory tract of infected ferrets, mice and non-human primates than seasonal influenza viruses^5–8^, and have continued to circulate as seasonal viruses. They retain known characteristic traits, including α-2,6-linked sialic acid receptor-binding specificity of HA, functional balance of HA and NA activities, and adaptation of polymerase to mammalian upper airway^9^. Activation pH has decreased during the evolution of pH1N1 from swine H1N1 via 2009 human pH1N1 to seasonal H1N1 isolates, revealing that HA stability is necessary for viral pathogenicity and transmissibility^10^.

HA precursor HA0 is synthesized and trimerized in the endoplasmic reticulum, which is transferred through the Golgi apparatus to the cell surface^11–13^. Cleavage of the precursor HA0 into HA1 and HA2 by a cellular protease is required for viral infectivity^14, 15^. HA is a metastable surface glycoprotein that undergoes large conformational changes at acidic pH^16, 17^. We previously showed that the purified recombinant HA ectodomain from a pH1N1 isolate, A/Korea/01/2009 (KR01), was a monomer in solution^18^. Those from A/California/04/2009 (CA04) and A/Darwin/2001/2009 (DA01) pH1N1s were also a monomer in solution, but their crystal structures showed a typical trimeric form^19, 20^, whereas the KR01 HA structure revealed a head-to-head arrangement of monomeric forms. The KR01 HA-Fab0757 complex structure also exhibited the head-to-head arrangement of monomeric HAs^18^. The HA sequence of KR01 differs at only three amino acid positions from that of CA04, Ser83(Pro83) [The amino acid residue is numbered according to KR01 HA and the corresponding amino acid residue of CA04 or DA01 HA is given in parenthesis.], Ala187(Thr187) and Val321(Ile321), and that of DA01, Ser203(Thr203), Arg205(Lys205) and Val411(Ile411), which are located far from the intermonomer interface or the site that might affect the monomer-trimer equilibrium. In contrast, the HA protein derived from A/Thailand/CU44/2006 (CU44), a seasonal strain, was a trimer in solution and crystal structure^18^. We noticed distinct amino acid sequence differences between the KR01 and CU44 HAs at the intermonomer interface: Ser199 in HA1, and Gly47 and Arg75 in HA2 in CU44, but Phe199, Glu47 and Leu75 in KR01.

Broadly neutralizing antibodies (bnAbs) against influenza viruses are mostly directed toward highly conserved conformational epitopes in the immunosubdominant HA stem^21^. Removal of the HA head and transmembrane domains without modifications to stabilize the remaining molecule led to loss of native conformations of the HA stem and consequently bnAb conformational epitopes^22^. Various strategies to enhance exposure of the HA stem to the host immune system have been attempted^23–29^. Anti-stem bnAbs were shown to mediate cytotoxicity of infected cells via Fc receptors^30^. However, stem-derived immunogens have often exhibited low affinity with bnAbs, indicating suboptimal conformations and low antibody titers. A recent analysis of serum antibody repertoires in young adults before and after seasonal influenza vaccination showed that broadly neutralizing cross-reactive antibodies bind to a monomeric HA at a highly conserved region occluded in HA trimer, which conferred full protection against influenza challenge^31^. This observation is contrary to that of earlier research showing that cross-reactive epitope sites require HA trimerization to attain full antigenicity. These HA trimer-dependent antibodies were nevertheless bound to monomeric HA with K_D_s in the range of nM, with slightly decreased affinity compared to the trimer^32^.

In this context, HA monomer that exposes the highly conserved intermonomer interface occluded in the trimer may be a good candidate for vaccine development, which is not available in the trimer and stem antigens. In addition, use of the self-assembling nanoparticle scaffold fused with the trimeric HA ectodomain or stem is currently constrained to the three-fold symmetry region, due to the trimeric nature^25, 28^. A stable HA monomer will provide an additional opportunity to develop various types of scaffold-based antigens. We thus aimed to generate a soluble HA monomer by introducing mutations at the intermonomer interface to destabilize the trimeric CU44 HA and examined the immunogenicity of HA monomer compared to that of the wild-type trimer *in vivo*.

## Results

### Preparation, purification and characterization of HA mutant monomers

**Figure 1:**
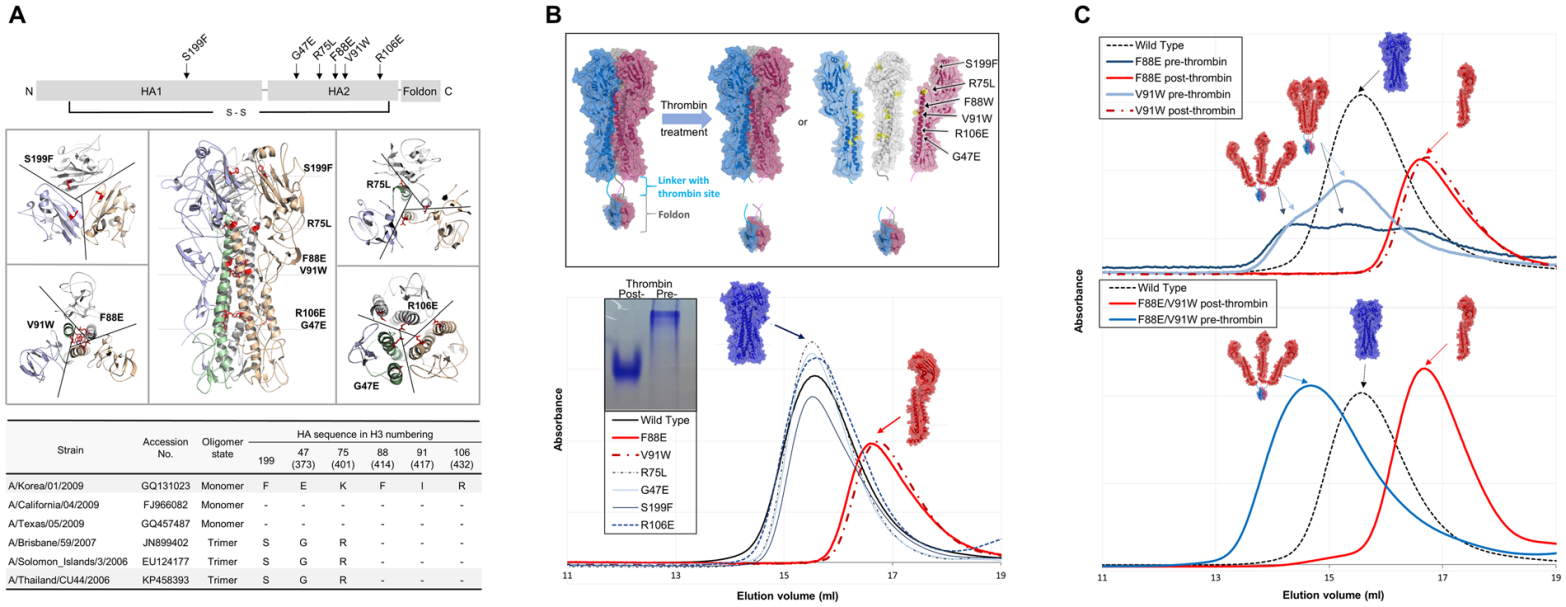
Modulation of the monomer-trimer equilibrium of HA. (**A**) The cloned HA construct with HA1, HA2 and the foldon, showing mutation sites with arrows and a disulfide bond between HA1 and HA2 (upper panel). The mutation sites are highlighted in red on the crystal structure of CU44 HA at the center with cross-section views along the dashed lines at the left (S199F, V91W and F88E) and right (R75K, R106E and G47E) (middle panel). The amino acid sequence differences at the mutation sites among 2009 pH1N1 and seasonal H1N1 HA proteins are shown (lower panel), where the amino acid residue is numbered according to KR01 HA and the corresponding amino acid residue of HA0 is given in parenthesis. The CU44 wild-type HA in this study showed 3 mutations different from the original CU44 HA: HA1 M116I and HA2 I91V and N169S. (**B**) Schematic diagrams showing a modulation of the monomer-trimer equilibrium upon the removal of the foldon by thrombin treatment (upper panel). The equilibrium can depend on mutation at the intermonomer interface. The HA trimer where each monomer is shown in blue, red and gray color is dissociated into monomers, if the mutation at the intermonomer interface induces either electrostatic or steric repulsion to destabilize the trimer. Characterization of HA mutant proteins using site exclusion chromatographic analysis (lower panel). F88E and V91W mutants were eluted as a monomer, whereas other mutants were eluted as the wild-type HA trimer. Native PAGE is shown in inset for the pre- and post-thrombin F88E mutant HA. (**C**) Characterization of the single mutant, F88E and V91W, and the double mutant F88E/V91W using size exclusion chromatography (upper and lower panels, respectively). Single and double mutants without the foldon (post-thrombin) were eluted as monomers. The wild-type HA trimer in blue is shown as a single peak (dashed line).

Pairwise sequence alignment showed 79.3% sequence identity between KR01 and CU44 HA proteins, which drops to 50.0% within the antigenic sites (Supplementary Fig. S1). Given that the recombinant KR01 HA ectodomain was a monomer in solution, we noticed amino acid sequence differences at the intermonomer interface between the KR01 and CU44 HAs (Fig. 1A): Ser199 of HA1 and Glu47(Glu373)^*^ and Lys75(Lys401) of HA2. Starting from the wild-type HA trimer of CU44, six mutants were constructed: S199F of HA1 and G47E of HA2, according to the corresponding amino acid of KR01 HA, and R75L, F88E, V91W, and R106E of HA2 in the stem region, in an attempt to destabilize the trimer due to electrostatic or steric repulsion. We prepared these mutants, fused to a foldon domain to promote folding and trimerization, with a thrombin cleavage site and 6xHis-tag at the C-terminus^33^. After removal of foldon using thrombin, we characterized the oligomerization states of the purified mutants by SEC and native PAGE (Fig. 1B). Four mutants were trimers upon removal of the foldon, like the CU44 wild-type HA, whereas two mutants, F88E and V91W, were detected as monomers in solution.

Both mutants in the presence of the foldon revealed several peaks on SEC (Fig. 1C, upper panel). F88E existed as monomers and trimers, whereas a trimer was dominant in V91W. One of the trimers of both mutants could be a loosely-assembled one due to electrostatic or steric repulsion. It appears that F88E has a higher tendency to destabilize the trimer than V91W, revealing higher proportions of the loosely-assembled trimer and monomer simultaneously. It is also possible that the peak corresponding to the loosely-assembled trimer can be generated by higher oligomeric forms due to the mutation. When the foldon was removed by thrombin cleavage, however, both mutants were eluted mainly as a single peak, indicating that the mutant ectodomains are predominantly a monomer in solution (Fig. 1C, upper panel). Our results thus demonstrate that the two CU44 HA mutants, F88E and V91W, become monomers upon proteolytic cleavage of the foldon.

### Characterization of HA double mutant

**Figure 2:**
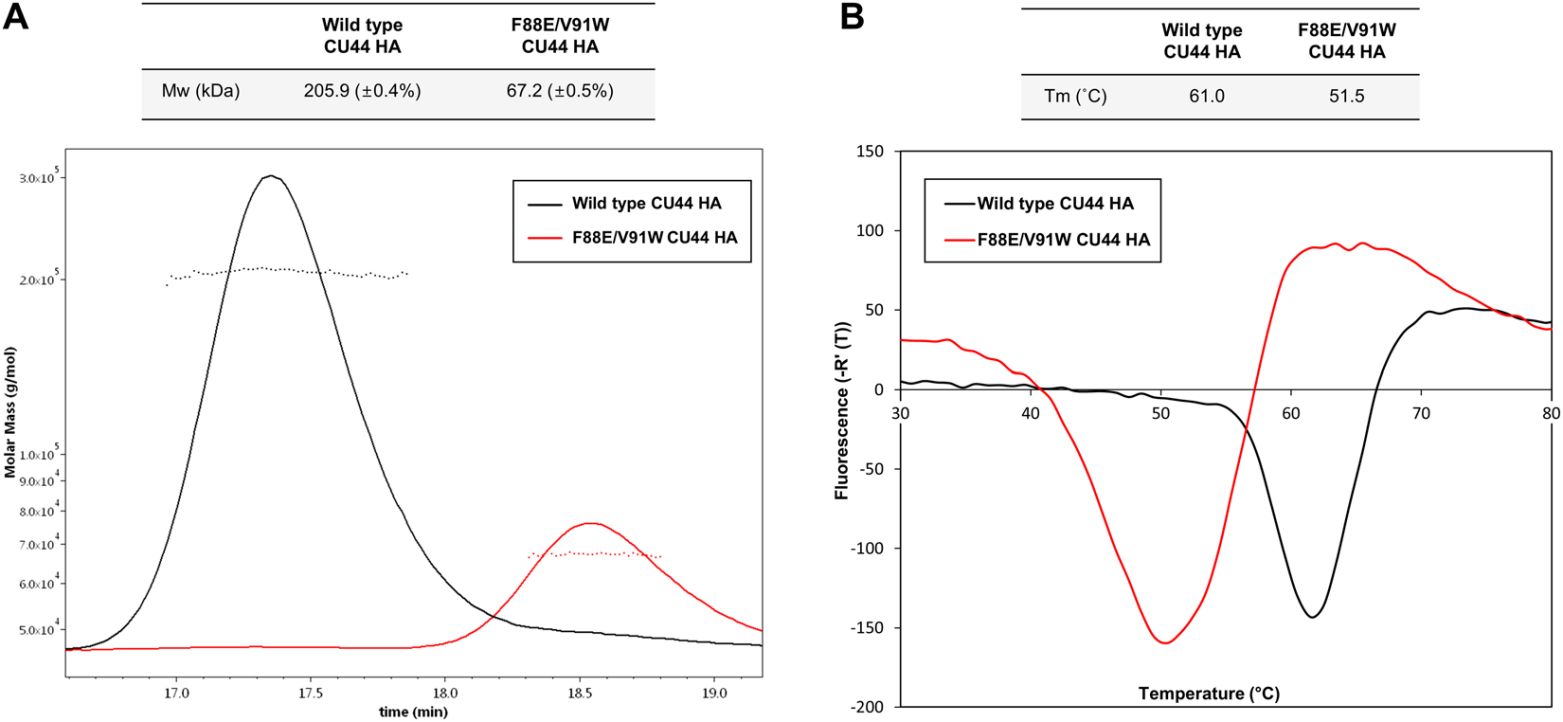
Characterization of HA mutant protein. (**A**) SEC-MALS analysis of the purified double mutant. The molecular masses of the CU44 wild-type and double mutant HA proteins agreed with the value of 67.2 kDa and 205.9 kDa, respectively, taking into account glycosylation. The flow rate in SEC-MALS was 0.5 ml/min using a UFLC system. (**B**) Differential scanning fluorimetry transition curves of the wild-type HA trimer (in black) and double mutant monomer (in red). Each HA protein was incubated at 25 °C for 30 sec, and then the temperature was increased by 0.5 °C every 30 sec for 50 min.

In order to further destabilize the HA trimer, we produced the double mutant F88E/V91W (Supplementary Fig. S2). As expected, the purified double mutant was eluted predominantly as a trimer and monomer before and after the foldon cleavage, respectively, based on SEC (Fig. 1C, lower panel). Interestingly, the double mutant was eluted mainly as a loosely-assembled one prior to the cleavage. The molecular mass of the double mutant deduced from the nucleotide sequence, including glycosylation, was 62.5 ( ± 0.1) kDa determined by SEC-MALS, which agreed with the value of 67.2 kDa upon post-cleavage in solution (Fig. 2A). In comparison, the molecular mass of the CU44 wild-type HA was 205.9 kDa, taking into account glycosylation. Our results thus demonstrate that the double mutant behaves as a monomer in solution and mutations that affect the intermonomer interface indeed destabilized the trimer form of the CU44 HA.

Previously, it was shown that removal of the HA head domain led to loss of native conformations of the stem region and concomitant loss of conformational bnAb epitopes^22^. In order to assess whether the mutations at the intermonomer interface can impact the stability of HA, the differential scanning fluorimetry (DSF) was used to measure the melting temperature T_m_ of the double mutant. It was found to have lower T_m_ (~10 °C) than the wild-type trimer (Fig. 2B), indicating that the monomer has a much lower stability than the trimer. The KR01 HA that was a monomer in solution had also lower T_m_ (5 °C) than the CU44 HA^18^.

### Immunogenicity of the HA double mutant monomer

**Figure 3:**
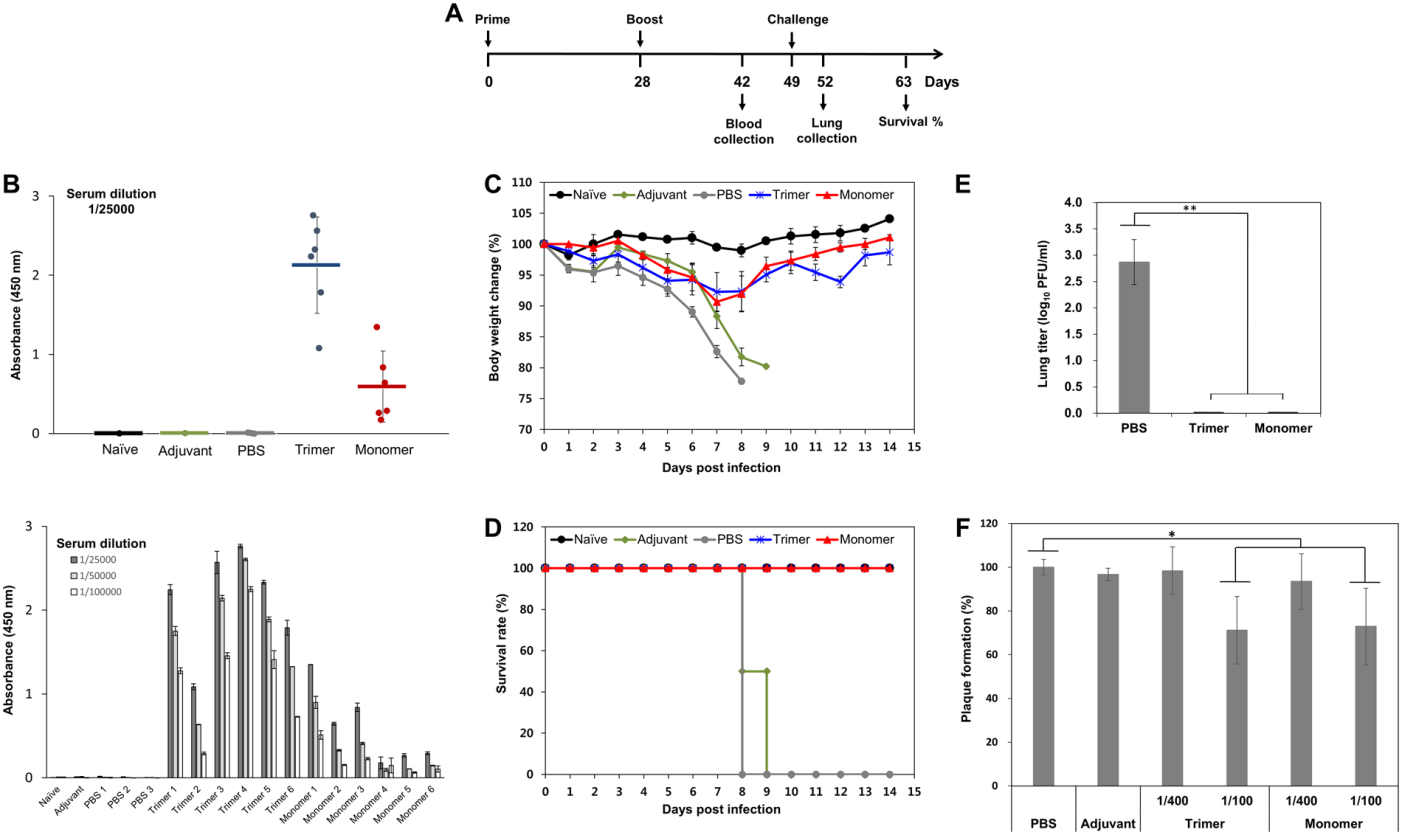
Protection of mice against virus infection by HA monomer. (**A**) Schedule of mouse immunization, sample collection and infection. BALB/c mice were immunized subcutaneously with 20 µg of recombinant HA antigen. Four weeks later, the mice were boosted with the same amounts of HA antigen. The groups were naive, wild-type trimer, mutant monomer, PBS control, and adjuvant control. After three weeks of the boost, the mice were challenged intranasally with mouse-adapted PR8 in PBS (3 MLD_50_). (**B**) IgG antibody titration from sera specific to HA antigens at a dilution of 1/25000 (upper panel) and at two-fold dilution starting with a dilution of 1/25000 (lower panel) were evaluated by ELISA. At day 42 that is at 2 weeks after the boost, the blood from mice of all groups were analysed for antibody response. Each individual dot represents the value from a single mouse, and the horizontal line represents the median value for the group. (**C**) Body weight changes and (**D**) survival rate of PR8-challenged groups. The percentages of body weight changes and survival rates were monitored daily until 14 dpi. Body weight data are the means ± SD for six mice per group ± SD. (**E**) Viral lung titers by PFU/ml at 3 dpi in BALB/c mice infected with PR8 virus. Values represent the means ± SD and statistical significance was defined as ^**^p < 0.01. (**F**) Plaque reduction neutralization assay. Serum samples were initially diluted at 1:10 and 1:40, and PR8 virus was added to each diluted serum sample tubes at the volume ratio of 9:1. The virus-serum mixture was incubated for 60 min and added to the MDCK cells at 37 °C and 5% CO_2_ for 60 min. The presence of virus–infected cells was indicated by the formation of viral plaques, and PR8-infected cells with PBS and adjuvant were used as controls. ^*^p < 0.05.

In order to examine whether the change in oligomeric states of HA can affect its immunogenicity, BALB/c mice were immunized subcutaneously with 2 doses of the antigen (20 μg/mice per dose) containing the HA double mutant monomer or wild-type trimer antigen, given at intervals of 4 weeks, following the schedule of a prime and boost illustrated in Fig. 3A. Intranasal challenges with mouse-adapted influenza virus PR8 at 3 weeks after the boost and ELISA for total IgG serum antibody responses at 2 weeks after the boost were performed. Subcutaneous administration of the HA monomer antigen yielded antibody titers significantly lower than that of the trimer. The serum antibody responses from the monomer varied greatly among individual mice but was much higher than those in the control (Fig. 3B, upper panel). IgG antibody titration from sera specific to HA antigens at two-fold dilution starting with a dilution of 1/25000 also showed the immunity by the monomer was comparable to that by the trimer (Fig. 3B, lower panel). When the antibody titration was examined against H3N2 A/Gyeongnam/684/2006 (Gy684), the serum antibody responses from the monomer were lower in general than those from the trimer (Supplementary Fig. S3). However, it was notable that monomer 3 yielded significantly higher antibody titers than those induced by the trimers. The trimer and monomer-treated mice did not show significant body weight loss when challenged with PR8. They lost only a maximum of 8–10% of their body weights at 7 dpi and their body weights returned to 99–101% at 14 dpi, showing full protection against virus challenge. However, mice in the negative control group started to show a clear body weight loss after 5 dpi (Fig. 3C). Infected mice immunized with PBS and adjuvant succumbed to death at 8 dpi and 9 dpi, respectively. Mice immunized with HA trimer or monomer survived a challenge with PR8 virus (Fig. 3D).

Notably, virus titers in the lungs of immunized mice were lower than those of non-immunized mice (Fig. 3E). In PR8-challenged mice, the virus titers were significantly reduced by about 3 orders of magnitude at the immunization with 20 μg/mouse HA monomer or trimer antigen. No virus was detected in the groups treated with either the HA monomer or trimer antigen, which demonstrates that the recombinant monomer antigen could decrease influenza virus distribution in the lungs as effectively as the trimer. Next, plaque reduction neutralization assays were performed using serum samples. Both serum samples induced by either the trimer or monomer were found to reduce plaque formation by 30% at 1:100 dilution (Fig. 3F). The monomer-induced serum sample showed neutralization activity comparable to the trimer-induced serum. Taken together, the protection was evident from the HA monomer, despite the low antibody titer, by increased survival and significantly reduced viral titers from the challenge.

## Discussion

The occurrence of influenza epidemics originated from antigenically highly diverse viruses is unpredictable. One of the most effective ways to protect against the diverse influenza virus infection is by vaccination. However, a good match between the epidemic and vaccine strains is difficult to attain due to the high plasticity of HA and consequent rapid antigenic drift to evade existing antibody responses^31^. Continuous improvement of vaccine strategies is thus important to ensure to provide broader and long-term immunity against influenza viruses. Cross-reactive bnAbs discovered recently are mainly directed toward highly conserved conformational epitopes in the HA stem. Various strategies to enhance exposure of the HA stem to the immune system have been explored: headless, chimeric and hyperglycosylated HAs and scaffold and virus-like particle-based HAs^21, 23–29^, to generate a stable and broadly protective HA stem immunogen. However, immune responses against the stem domain of HA were weak relative to those against the head domain, and the quest to efficiently elicit bnAb titers against homologous and heterologous influenza viruses has remained challenging^21, 32^. Despite their close packing on the viral membrane, the stem domains of HA trimers on the surface of the virus are nevertheless accessible to the stem-based bnAbs^34^.

Recently, cross-reactive bnAbs in serum were found to bind to HA monomer rather than HA trimer^31^. None of the bnAbs showed hemagglutination inhibition activity and half of them showed no neutralization activity in cell-based assays. However, they showed full protection against influenza challenge in mice. Interestingly, when the Fab fragments were mixed with the trimeric HA molecules, dissociation of the HA trimer was observed to form a 1:1 complex of one monomer with one Fab molecule. In order to understand whether the epitope on HA monomer can be immunogenic and valuable in vaccine development, HA monomer was prepared in this study by destabilizing the intermonomer interface of the wild-type trimer of CU44 HA. Among six mutants, R106E was designed to reduce intermonomer interactions by electrostatic repulsion, where negatively charged amino acids, Glu103, Glu105 and Asp109, are positioned very close at the stem region. However, the replacement of Arg106 with Glu106 was not successful to destabilize the trimer. The S199F, G47E and R75L mutants also did not affect the monomer-trimer equilibrium. In contrast, two mutants, F88E and V91W, were identified as a monomer in solution upon cleavage of the foldon, by disrupting intermonomer interactions of HA. The F88E/V91W double mutant also behaved as a monomer, which appeared to be more homogeneous than the single mutants. However, the double mutant monomer showed less stability than the trimer, with a T_m_ of 10 °C lower than that of the CU44 HA trimer. It was reported that the recombinant KR01 HA was a monomer in solution and less stable than the CU44 HA trimer^18^. The double mutant also seems to have a lower stability in thermal denaturation experiments than the KR01 HA, possibly due to the introduction of two additional mutations (F88E and V91W) at the intermonomer interface.

Despite the significant difference in stability between monomeric and trimeric HA proteins, the *in vivo* administration of the monomer enhanced protection against PR8 infection, comparable to that of the HA trimer. It showed significant body weight gains and significant reduction of lung titers in PR8-challenged mice. In recent studies of stem-based bnAbs with protection from mortality, they failed to reduce lung viral loads^28, 29^. In this respect, the double mutant offers potential protection against H1N1 viruses and the mode of protection is related to limiting virus replication in the lung. However, the HA monomer antigen induced antibody titers that varied among individual mice and were significantly lower than the trimer, although much higher than those in the control. The KR01 HA showed a monomer in the crystal structure, where a part of HA2 was not visible, indicating the monomer is more flexible, compared to the trimer^18^. It is possible that a typical HA has a hinge motion between the head and stem regions, and the comparison between KR01 and CU44 HA monomer structures strongly suggest that the hinge motion in HA is more prominent in the monomer than in the trimer. We postulate that the double mutant monomer adopts a flexible and less stable conformation, compared to the trimer. Nevertheless, we cannot rule out the possibility that the stem region in the monomer can be aggregated or misfolded, as was shown in the group 1 HAs that form high molecular weight multimers, when it was lack of the foldon^35^.

Protein stability may help to limit protein aggregation, and the HA monomer has a high tendency to aggregate (data not shown). In a recent study, formation of an intermonomer disulfide bridge in mini-HA trimer contributed to increased conformational stability^29^. A broad and extremely potent HIV-specific monoclonal antibody was shown to bind to a HIV-1 Env epitope, by engineering a stable and soluble Env trimer^36, 37^. We attempted to enhance the stability of the mutant monomer by disulfide formation that can stabilize the hinge motion. However, it was not successful, mainly due to the lack of appropriate geometry to form a disulphide bond between HA1 and HA2 at the hinge region. Instead, the hydrophobic amino acid residues that are hidden at the intermonomer interface become exposed on the surface of the monomer, which we plan to replace with hydrophilic ones.

Another possibilities to account for the low antibody titers by the HA monomer can be explained by the quantity and specificity of serum antibodies. The HA antigen in group 1 influenza viruses is known to possess five distinct antigenic sites in the head region: Sa, Sb, Ca1, Ca2, and Cb. Full epitope antigenicity at sites Ca, Cb and Sb was known to require HA trimerization and it persisted even after proteolytic release of monomeric heads^32^. We speculate that the monomer can present the intermonomer interface that is hidden in the interior of the trimer. HA monomer-specific antibodies may thus bind to the monomer antigen more significantly than the trimer, when the access to the hidden region at the trimer is critical. It was reported that neutralizing antibody titers induced by stem-based antigens were low in animal models, which appeared to be common^24, 28, 29, 38^. Cross-reactive antibodies that provide protection *in vivo* often showed little neutralizing activity *in vitro*, suggesting non-neutralizing protection^39–41^. In this context, it would be interesting to examine the epitope specificity at the intermonomer interface or stem region of the monomer.

In conclusion, the HA double mutant as a monomer showed immunogenicity comparable to that of trimeric HA, albeit low stability. The immune protection with the monomer in mice was encouraging for the development of a stable monomer immunogen. The HA monomer in this study was obtained by destabilizing the HA trimer and further investigation will be focused to enhance the stability of the HA monomer. Development of the monomer HA with higher stability will be technically challenging, given that the HA stem region is known to be unstable. Nevertheless, monomer HA will be the first step towards an immunogen that elicit antibodies effective against diverse strains of influenza virus.

## Methods

### Preparation of cells

MDCK cells (American Tissue Culture Type CCL-34) were grown in Dulbecco’s modified Eagle’s medium (DMEM: Gibco BRL, Karlsruhe, Germany) supplemented with 10% fetal bovine serum (FBS, Sigma-Aldrich, St. Louis, MO, USA) and 1% penicillin-streptomycin (Gibco BRL) under 5% CO_2_ humidified atmosphere at 37 °C. The cells were passed for no more than 20 passages.

### Virus collection and plaque assay

MDCK cells were seeded at 2 × 10^5^ cells/well in 24-well tissue culture plates (Corning Costar, Corning, NY, USA) and incubated for three days at 37 °C and 5% CO_2_ until approximately 95% confluency was reached. Influenza H1N1 virus, A/Puerto Rico/8/1934 (PR8), was propagated in MDCK cells with DMEM supplemented with 2 μg/ml of L-tosylamide-2-phenylethyl chloromethyl ketone (TPCK)-treated trypsin (Sigma-Aldrich) at 5% CO_2_ and 37 °C for 2–3 days. Virus titers (plaque forming unit (PFU)/ml) were determined by using plaque assay and virus stocks were stored in deep freezer (−80 °C) until experiments.

### Plaque assay

MDCK cells were infected with PR8 in a 24-well plate at 37 °C and 5% CO_2_ for 60 min. The inocula were removed and the cells were washed twice with PBS and overlaid with DMEM containing 1 µg/ml TPCK-treated trypsin and 1% agarose at 37 °C and 5% CO_2_ for 72 h. Plaques were counted after 0.5% crystal violet staining. The titer of the negative control serum sample remained negative.

### Cloning and baculovirus production of HA antigens

H1N1 A/Thailand/CU44/2006 (CU44) and H3N2 A/Gyeongnam/684/2006 (Gy684) HA proteins were produced in insect cells using recombinant baculovirus expression vectors. The synthesized cDNA of CU44 wild-type and Gy684 HA after RNA extraction was amplified using polymerase chain reaction (PCR). Wild-type HA gene (1–503 of HA0 based on H3 numbering) was cloned in pFastBac^TM^HT A (Invitrogen, Carlsbad, CA, USA) downstream of the gp67 secretion signal sequence of the transfer vector pAcGP67A (BD Biosciences, Woburn, MA, USA). The cloned construct had residues 11–329 (1–327) (HA1) and 330–506 (1–176) (HA2), with a thrombin cleavage site, foldon domain, and 6xHis-tag downstream of the HA gene sequence. The recombinant HA protein contained additional plasmid-encoded residues ADPG and RSLVPR at the N- and C-termini, respectively. Mutant HA genes were prepared by site-directed mutagenesis. Plasmids encoding each mutant HA gene (S199F of HA1 and G47E, R75L, F88E, V91W, R106E, and F88E/V91W of HA2) was amplified in *Escherichia coli* strain DH5α and the recombinant bacmid was generated according to the Bac-to-Bac expression system protocol (Invitrogen). All sequences were confirmed by automated sequencing (Macrogen, Seoul, Korea). The CU44 HA in this study showed 3 mutations different from the original CU44 HA: HA1 M116I and HA2 I91V and N169S, which was however used as the wild-type and these mutations also exist in nature. *Spodoptera frugiperda* (Sf-9) insect cells were transfected with the recombinant bacmid using Cellfectin II (Invitrogen). The transfected cells were harvested at 72 h post-transfection and centrifuged at 2,000 rpm for 15 min to get the baculovirus, expressing recombinant HA in the supernatant, and transfection efficiency was confirmed by PCR after extraction of DNA from 400 μl of virus. Virus titer was determined by plaque assay.

### HA antigen expression and purification

Baculovirus containing CU44 HA wild-type or mutant gene was used to infect suspension cultures of High Five cells. After 3 days at 28 °C, the culture medium was harvested. After centrifugation at 4,000 rpm for 30 min, the supernatant was applied to a nickel-nitrilotriacetic acid (Ni-NTA) affinity column (Qiagen, Hilden, Germany) equilibrated with 20 mM Tris-HCl, pH 8.0, and 100 mM NaCl. The column was washed with buffer containing 30 mM imidazole and precursor HA protein was eluted in an imidazole gradient (30–400 mM), which was dialysed against 20 mM Tris-HCl (pH 8.0) and 20 mM NaCl, and hydrolysed by thrombin for 4–5 h at 4 °C for removal of the foldon and 6xHis-tag. The precursor form of HA was purified by mono Q ion-exchange chromatography (GE HealthCare, Uppsala, Sweden) and Superdex 200 10/300 GL size exclusion chromatography in 50 mM Tris-HCl (pH 8.0) and 100 mM NaCl, connected to an ÄKTA FPLC system (GE HealthCare). It was concentrated in an Amicon 10,000 MWCO concentrator (Merck Millipore, Billerica, MA, USA) to 1–2 mg/ml for characterization and immunization. The recombinant Gy684 HA for ELISA was also expressed and purified according to the protocols for the CU44 HA.

### Native polyacrylamide gel electrophoresis (PAGE)

The purified proteins were prepared under native condition (mixed with 5x loading buffer, 312.5 mM Tris-HCl (pH 6.8), 50% glycerol and 0.05% bromophenol blue, without β-mercaptoethanol and sodium dodecyl sulphate). The electrophoresis was carried out in 25 mM Tris-HCl buffer (pH 8.8) with 192 mM glycine at 200 V and 80 mA for 1 h. The gels were stained using Coomassie Brilliant Blue R-250 (Sigma-Aldrich).

### Differential scanning fluorimetry (DSF)

DSF was used to measure the shift in transition temperature upon ligand binding, using a Stratagene MX3005 P (Agilent Technologies, Santa Clara, CA, USA). 25 µl of reactions containing 5 µg of HA wild-type or mutant, 5X SYPRO Orange (Invitrogen), 50 mM Tris-HCl (pH 8), and 100 mM NaCl were incubated at 25 °C for 30 s. Temperature increment at the rate of 0.5 °C every 30 s for 50 min and relative fluorescence units were recorded at the excitation and emission wavelengths of 492 nm and 610 nm, respectively. Transition temperature was calculated from the maxima of the first derivative of relative fluorescence units/temperature using MxPro QPCR Software.

### Size exclusion chromatography with multi-angle light scattering (SEC-MALS)

The shift in elution volume of the mutant monomer was compared to that of the wild-type HA, which was determined by size exclusion chromatography (SEC) on a Superdex 200 GL column (GE HealthCare) equilibrated with 50 mM Tris (pH 8.0) and 100 mM NaCl at a flow rate of 0.5 ml/min. To determine the molecular weight change of the HA mutant protein, purified protein (100 µl) was applied to GPC PSS PROTEEMA (PSS, Mainz, Germany) analytical column at 0.5 ml/min using a UFLC system (Shimadzu, Kyoto, Japan). Light scattering and refractive index were measured using in-line WYATT-787-TS miniDAWN TREOS (Wyatt Technology, Santa Barbara, CA, USA), which were analysed by Astra 6 software.

### *In vivo* mouse experiments

All animal experiments were performed in accordance with the recommendations in the Guide for the Care and Use of Laboratory Animals from the Animal, Plant and Fisheries Quarantine and Inspection Agency, Republic of Korea. The study protocol was approved by the Institutional Animal Care and Use Committee of Duksung Women’s University. All efforts were made to reduce suffering of the animals which were kept under specific pathogen-free conditions and sacrificed upon a body weight loss of 20% at the utmost. Groups of six female BALB/c mice (Koatech, Namyangju-si, Kyunggi-do, Korea) at five weeks of age were adapted for a week and immunized subcutaneously with recombinant HA antigen, trimer or monomer. The trimer or monomer groups received 20 µg of the recombinant HA in 200 µl of PBS (pH 7.4) with adjuvant system (SAS) (Sigma-Aldrich). Four weeks later, the mice were boosted with the same amounts of HA antigens. Blood samples were collected at two weeks after the boost. At three weeks after the boost, the mice were challenged intranasally with 3 MLD_50_ of mouse-adapted A/Puerto Rico/8/1934 (PR8) virus. Body weights and survival rates were monitored daily until 14 days post infection (dpi). Mice were sacrificed at 3 dpi to determine lung virus titers, which were determined by plaque assay. For virus lung titers, homogenates of the lung tissue were centrifuged at 4,000 × *g* and 4 °C for 5 min. The supernatant was collected to be lung suspension. MDCK cell monolayers were infected at 37 °C for 3 h with 500 μl of the lung suspension in 1:10 dilution in a 24-well plate. After infection, cells were washed and overlaid with DMEM containing 1 μg/ml TPCK-treated trypsin and 1% agarose for 72 h at 37 °C in a 5% CO_2_ incubator. Plaques were counted after 0.5% crystal violet staining.

### Plaque reduction neutralization assay

Serum samples were initially diluted at 1:10 and 1:40 in the eppendorf tubes. PR8 virus was added to each diluted serum sample tubes at the volume ratio of 9:1, which was incubated for 60 min. MDCK cells were infected with the incubated serum sample-PR8 virus in a 24-well plate at 37 °C and 5% CO_2_ for 60 min. The inocula were removed and the cells were washed twice with PBS and overlaid with DMEM containing 1 µg/ml TPCK-treated trypsin and 1% agarose at 37 °C and 5% CO_2_ for 72 h. Plaques were counted after 0.5% crystal violet staining. The titer of the negative control serum sample remained negative.

### Enzyme-linked immunosorbent assay (ELISA)

The 8-well strip was coated with the CU44 or Gy684 HA proteins in a total volume of 100 µl (2 ng/µl) for ELISA (Corning) overnight at 4 °C. Following 1% of BSA blocking and PBS washing, primary antibody from diluted serum samples were bound for 1 h at 37 °C, washed with PBS and detected by anti-mouse IgG1-HRP (Abcam, Cambridge, UK) as the secondary antibody for 1 h at 37 °C. 150 µl of 3,3′,5,5′-tetramethylbenzidine (Sigma-Aldrich) was used as substrate and the reaction was stopped by addition of 2 N sulfuric acid, and absorbance at 450 nm was monitored.

### Statistical analysis

Plaque assays carried out in triplicate. Experimental results were expressed as the mean and SD. Data were analysed using analysis of variance (ANOVA) with SPSS software (version 13.0, SPSS Inc., Chicago, IL, USA), and the means were separated by Tukey’s multiple comparison test. Statistical significance was defined as follows: ^*^p < 0.05, ^**^p < 0.01.

## Electronic supplementary material

Supplementary information
