# Supplementary material for: Conformational modulation of influenza virus hemagglutinin: characterization and in vivo efficacy of monomeric form
Source: Sci Rep. 2017 Aug 8;7:7540. doi: 10.1038/s41598-017-08021-x (PMC5548806; doi:10.1038/s41598-017-08021-x)
Supplement: Supplementary file 1 — Supplementary information [file 41598_2017_8021_MOESM1_ESM.pdf]

Supplementary Materials

Conformational modulation of influenza virus hemagglutinin: characterization and in vivo efficacy of monomeric form

Jong Hyeon Seok[a], Jeongwon Kim[b], Dan Bi Lee[a], Ki Joon Cho[c], Ji-Hye Lee[a], Karam Bae[b], Mi Sook Chung[b], and Kyung Hyun Kim[a*]

[a]Department of Biotechnology & Bioinformatics, Korea University, Sejong 30019, Korea,

[b]Department of Food and Nutrition, Duksung Women's University, Seoul 01369, Korea.

[c]Antibody Engineering Team, Mogam Institute, Yongin Kyunggi 16924, Korea

Supplementary information

Contents

Figures S1-S3

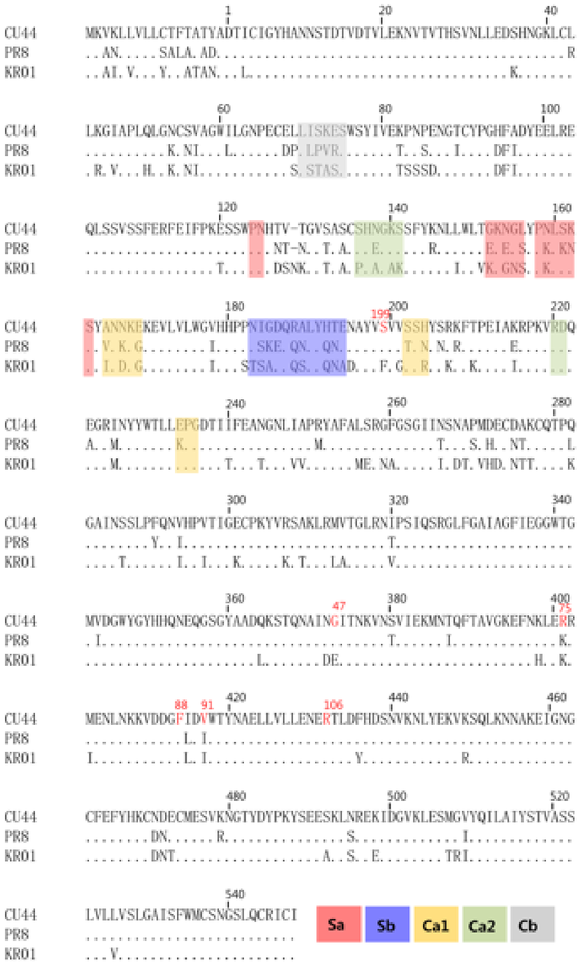

	CU44	PR8	KR01
Full length Identity (%)	100.0	86.2	79.3
Antigenic site Identity (%)	100.0	52.0	50.0

Fig. S1. Amino acid sequence alignment of influenza virus hemagglutinin proteins. Sequence alignment of CU44, PR8 and KR01 HAs in full lengths. Antigenic sites Sa, Sb, Ca1, Ca2 and Cb of H1 HAs were highlighted in pink, blue, yellow, green and gray, respectively. Six mutation sites (S199F, G47E, R75L, F88E, V91W and R106E) were marked in red. Sequence identities of the full length and antigenic sites between CU44, PR8 and KR01 HAs were calculated in the table on the right.

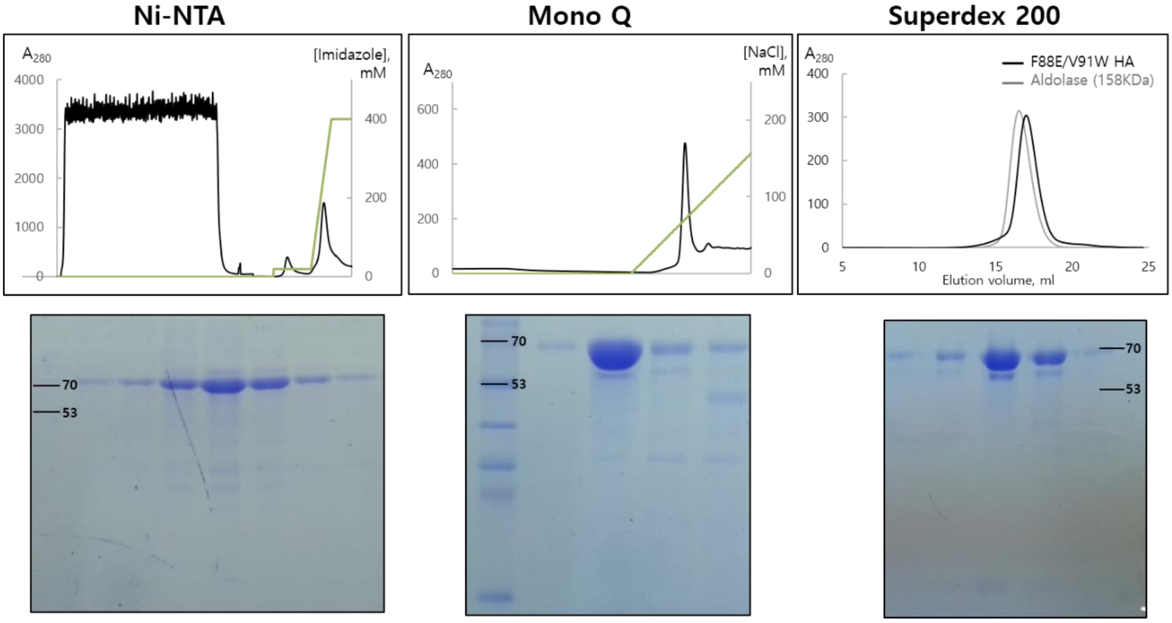

Fig. S2. Chromatographic elution profiles and SDS-PAGE results of the double mutant F88E/V91W HA during purification. The supernatant containing the F88E/V91W HA monomer expressed in baculovirus expression system was applied to Ni-NTA affinity column (left panel). After elution and thrombin treatment for removal of the fold and 6xHis-tag, the HA monomer was purified by mono Q ion-exchange chromatography (middle panel) and size exclusion chromatography using Superdex 200 column (right panel). Aldolase (158 kDa) was used as a control, which is eluted between CU44 wild type trimer (205.9 kDa) and double mutant monomer (67.2 kDa).

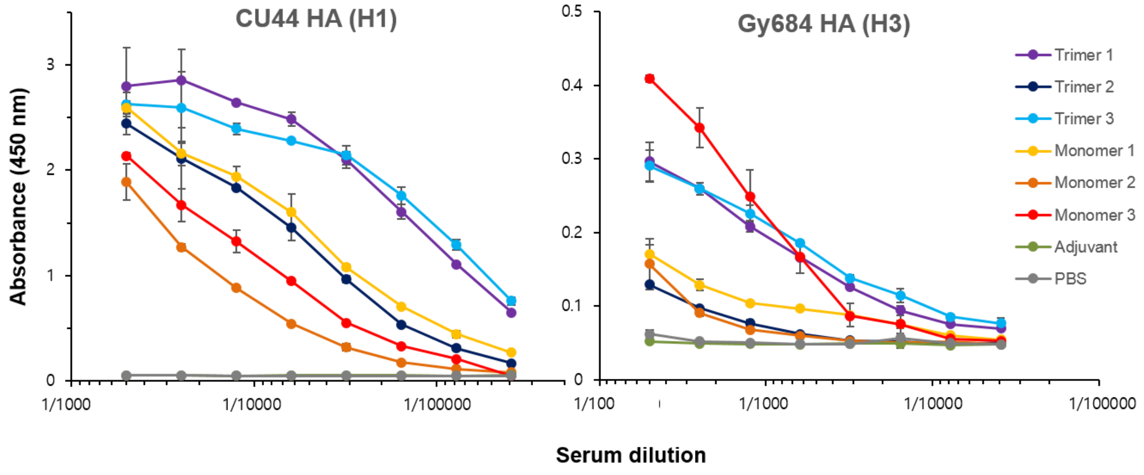

Fig. S3. IgG antibody titration curves from sera specific to HA antigens. Antibody titration was examined by ELISA against H1N1 PR8 (left panel) and H3N2 Gy684 (right panel) at two-fold dilution starting with a dilution of 1/2000 and 1/200, respectively. The serum antibody responses from the monomer were lower in general than those from the trimer, and monomer 3 yielded significantly higher antibody titers than those induced by the trimers.
